# COVID-19 and Health-Related Quality of Life: A Community-Based Online Survey in Hong Kong

**DOI:** 10.3390/ijerph18063228

**Published:** 2021-03-20

**Authors:** Edmond Pui Hang Choi, Bryant Pui Hung Hui, Eric Yuk Fai Wan, Jojo Yan Yan Kwok, Tiffany Hei Lam Tam, Chanchan Wu

**Affiliations:** 1 School of Nursing, LKS Faculty of Medicine, The University of Hong Kong, Hong Kong, China; jojoyyk@hku.hk (J.Y.Y.K.); wuchanchan@connect.hku.hk (C.W.); 2 Department of Applied Social Sciences, The Hong Kong Polytechnic University, Hong Kong, China; bryant-p-h.hui@polyu.edu.hk; 3 Department of Family Medicine and Primary Care, LKS Faculty of Medicine, The University of Hong Kong, Hong Kong, China; yfwan@hku.hk; 4 Department of Pharmacology and Pharmacy, LKS Faculty of Medicine, The University of Hong Kong, Hong Kong, China; 5 LKS Faculty of Medicine, The University of Hong Kong, Hong Kong, China; tiffthl@connect.hku.hk

**Keywords:** bother, COVID-19, health-related quality of life, health status, Hong Kong

## Abstract

The COVID-19 pandemic itself and related public health measurements have had substantial impacts on individual social lives and psychological and mental health, all to the detriment of health-related quality of life (HRQoL). There have been extensive studies investigating the mental health of people in different populations during the COVID-19 pandemic. However, few studies have explored the impact of COVID-19 and its association with HRQoL. To fill this research gap and provide further empirical evidence, this study examined the impact of COVID-19 on Hong Kong people and evaluated its association with HRQoL. A total of 500 participants were randomly recruited to complete an online questionnaire on their concerns related to COVID-19. This entailed responding to the World Health Organization Quality of Life-BREF instrument. Data were collected between 24 April and 3 May 2020. Independent t-tests and multiple linear regressions were used to examine the association between the impact of COVID-19 and HRQoL. Overall, 69.6% of participants were worried about contracting COVID-19, and 41.4% frequently suspected themselves of being infected. Furthermore, 29.0% were concerned by the lack of disinfectants. All of these findings were associated with poorer HRQoL in the physical and psychological health, social relationships, and environment domains. On the other hand, 47.4% of participants were concerned that they may lose their job because of the pandemic, while 39.4% were bothered by the insufficient supply of surgical masks. These two factors were associated with poorer HRQoL in the physical and psychological health and environment domains. The adverse impact of COVID-19 on individuals is multifactorial, affecting all aspects of HRQoL. In addition to enhancing anti-epidemic efforts, it is equally important to implement public health and social welfare measures, thereby diminishing the adverse impact of COVID-19 on overall well-being.

## 1. Introduction

In December 2019, the highly contagious coronavirus disease COVID-19 emerged in Wuhan City, China [1]. The initial outbreak rapidly evolved into an explosive global pandemic [2], affecting more than 180 countries and regions [3]. With the number of confirmed COVID-19 cases surging past 10 million globally as of 15 July 2020 [3], there seems to be no end in sight to the health crisis. Since learning of the viral outbreak in January 2020, the Hong Kong government has implemented precautionary measures to attenuate the spread of infection, such as physical distancing rules, mandatory quarantine, travel restrictions, and temporary closure of schools and certain entertainment establishments [4,5,6,7]. Although these preventative measures played a role in averting a major local epidemic [5], these tough controls, coupled with the health threats from COVID-19, may lead to detrimental effects on individuals’ health-related quality of life (HRQoL) [8].

The World Health Organization has conceptualized HRQoL as an individual’s perception of his or her health and health-related domains of well-being [9]. HRQoL is dynamic, subjective and multidimensional. The dimensions include physical, social, psychological and environmental factors [9,10]. HRQoL is an important clinical outcome because poor HRQoL is associated with adverse clinical outcomes. For example, a study among Scottish adults found that physical aspects of HRQoL could predict all-cause death, incident cancer and coronary heart disease [11]. A study of Spanish older adults also found that changes in HRQoL could predict mortality [12]. Another study also found that a decrease in HRQoL has been significantly associated with increased odds of negative outcomes, including the inability to work due to health, hospitalization at one year and mortality [13].

With respect to the psychological impact of COVID-19, it has been found that the viral outbreak has had a substantial effect on people’s mental health [4,14,15,16]. Apart from the fear of direct health threats, psychosocial stressors, such as severe disruptions to routines, shortages of daily necessities, overwhelming amounts of health information related to COVID-19, and uncertainty over the outbreak could all be associated with heightened levels of anxiety and depression [14,17,18]. Concerning social factors, the lack of face-to-face social interaction could compromise social well-being despite the use of digital communications. It is suggested that social isolation and loneliness could pose diverse health risks to individuals, and in particular, lead to lower resilience to stress [19], higher risk of depression [20], less effective immune responses [20], and a decline in cognitive function [21]. Amid the pandemic, border shutdowns, travel restrictions, and quarantine policies have caused widespread economic aftershocks [22]. Nearly all sectors, especially hospitality, aviation, and the tourism industry, have faced an unprecedented economic crisis, resulting in a record-high unemployment rate in Hong Kong. Accordingly, income instability could undermine the HRQoL of individuals [23].

For most Hong Kong people, the COVID-19 pandemic reminded them of the difficult time in 2003, when severe acute respiratory syndrome (SARS) took the lives of nearly 300 local citizens [24]. Hence, at the very beginning of the current pandemic, the public stocked up surgical marks, disinfectants, and other cleaning products as precautions. Together with the public health measures imposed by the local government, this had certainly affected people’s HRQoL. However, there is little information on this aspect to date [25], as existing studies have mainly focused on the impact of COVID-19 and its associations with depression, anxiety, and fear [4,26]. In view of this, to supplement previous studies and provide a more holistic view of the adverse impact of COVID-19, this study aimed to investigate the impact of the viral outbreak on Hong Kong people and to evaluate its association with HRQoL. Assessing HRQoL during the COVID-19 pandemic helps identify the impacts of the pandemic on people’s everyday lives. Therefore, clinicians, social workers and other healthcare professionals can develop appropriate psychosocial interventions to alleviate the impacts of the pandemic on people’s well-being and daily lives.

## 2. Materials and Methods

### 2.1. Study Design

This was a cross-sectional study.

### 2.2. Setting and Participants

This study was a community-based study, which was carried out in Hong Kong. To be eligible, study participants would need to be (i) currently living in Hong Kong, (ii) able to read and understand Chinese, and (iii) aged 18 years or older.

**Figure 1:**
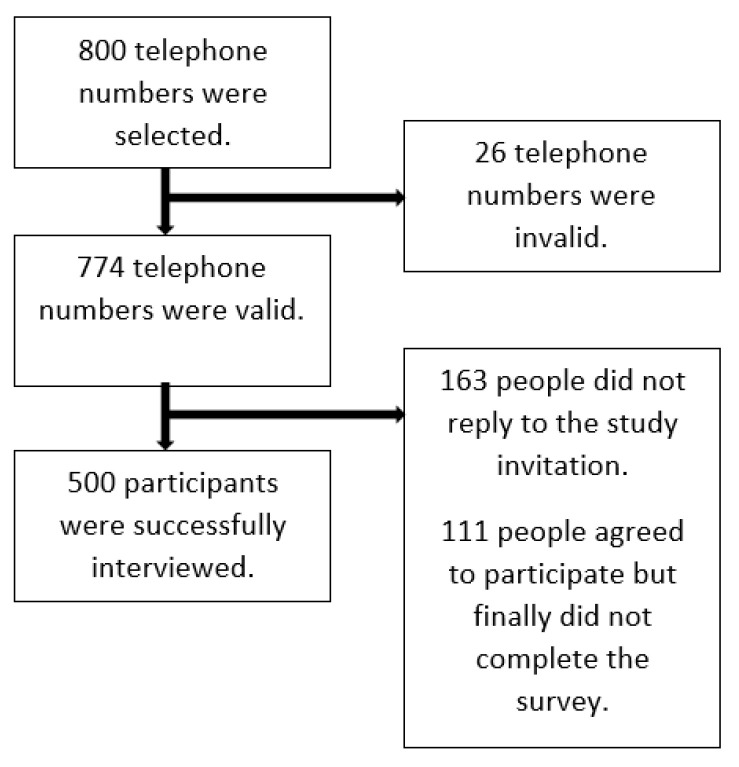
The flow diagram of recruitment.

A mobile phone-based random sampling approach was used to recruit study participants. The random list of mobile phone numbers was generated through the numbering plan for telecommunication services in Hong Kong provided by the Office of the Communications Authority. Mobile text messages were sent to randomly selected study participants to invite them to take part. After they had agreed to complete the questionnaire study, a link to a self-administered online survey was sent to them. The data collection period was between 24 April and 3 May 2020 in Hong Kong, which was approximately three months after the first local COVID-19 case was reported. Figure 1 shows the flow diagram of recruitment.

### 2.3. Study Instruments

The World Health Organization Quality of Life-BREF (WHOQOL-BREF) was used to measure the generic HRQoL in the study [27]. The WHOQOL-BREF consists of 24 questions in four domains, namely physical health (seven items), psychological health (six items), social relationships (three items), and environment (eight items). Each question w rated on a 5-point Likert scale. The scores within each domain are averaged. These average domain scores are multiplied by 4 to obtain transformed scores on a scale of 4–20 according to the guideline of the study instrument. Finally, the transformed domain scores are transformed linearly to a 0–100 scale [28,29,30], with a higher score indicating better HRQoL. The instrument has been validated among Chinese individuals living in Hong Kong [31]. Permission to use this instrument was obtained from the World Health Organization.

To assess the impact of COVID-19 on individuals’ HRQoL, the following statements were developed for the present study:

(i) I feel worried that I will be infected with COVID-19;
(ii) I feel worried that my family members will be infected with COVID-19;
(iii) I feel bothered because I often suspect that I have COVID-19 symptoms;
(iv) I feel bothered because I do not have enough surgical masks;
(v) I feel bothered because I do not have enough disinfectant supplies at home;
(vi) I feel worried that I may lose my job because of COVID-19.

The recall period for these five statements was two weeks, and a 4-point Likert scale was used (0 = not at all, 1 = several days, 2 = more than half the days, and 3 = nearly every day).

These items about the impact of COVID-19 were developed by the investigation team based on the pandemic situation from January 2020 to March 2020 in Hong Kong. The items were piloted on five people before we rolled out the study.

Participants also had to answer questions on sociodemographics, including gender, age, marital status, educational attainment, employment status, and income.

### 2.4. Statistical Analysis

First, descriptive statistics, such as mean and standard deviation (*SD*), were used to summarize the domain scores of the WHOQOL-BREF as well as sociodemographic characteristics. Second, we dichotomized the responses of the 6 statements related to COVID-19. Study participants who answered “0 = not at all” were coded as “no”, while study participants who answered “1 = several days”, “2 = more than half the days”, or “3 = nearly every day” were coded as “yes”. Independent *t*-tests were used to compare the mean HRQoL score of four domains between two groups (“yes” vs. “no”). Cohen’s *d* was also calculated, which classifies effect sizes as small (*d* = 0.2), medium (*d* = 0.5), and large (*d* = 0.8) [32]. Third, multiple linear regression analysis was used to test the robustness of the relationship between the impact of COVID-19 and the four domains of HRQoL after controlling for the sociodemographic factors.

The program Statistical Package for the Social Sciences (version 25) was used for data analysis. *p*-values < 0.05 indicated statistical significance.

### 2.5. Ethics

Ethics approval was obtained from the Institutional Review Board of the University of Hong Kong/Hospital Authority Hong Kong West Cluster (HKU/HA HKW IRB). Reference: UW20-217.

Electronic informed consent was obtained for each participant.

## 3. Results

### 3.1. Sample Characteristics

The mean age was 47.26 years (*SD* = 15.82), with 274 (54.8%) females and 226 males. 336 participants (67.2%) were currently married, and 281 participants (56.2%) had a full-time job. There were 159 participants (31.8%) with a bachelor’s degree or above, while 339 participants (67.8%) had a monthly personal income less than Hong Kong Dollar (HKD$) 20,001. Table 1 presents the sociodemographic characteristics of the study participants.

### 3.2. Impact of COVID-19

Overall, 348 participants (69.6%) were worried about contracting COVID-19, while 371 participants (74.2%) were worried that their family members would be infected by COVID-19. We found that 207 participants (41.4%) often suspected themselves of being infected. Furthermore, 197 participants (39.4%) feared that they would run out of surgical masks, and 145 participants (29.0%) were bothered by not having enough disinfectant supplies at home. Finally, 237 participants (47.4%) were concerned that they might lose their job because of COVID-19. Table 1 presents the descriptive statistics for the impact of COVID-19 on study participants.

### 3.3. HRQOL Scores

Compared with participants who at ease with the risk of infection, those who worried about contracting COVID-19 had poorer HRQoL across the four domains. We also found that those who worried that their family members would be infected with COVID-19 had poorer physical aspects of HRQoL only.

For participants, who often suspected themselves of being infected, and therefore, feeling worried had poorer HRQoL across the four domains than those who were not. Participants who were bothered by the insufficient supply of surgical masks had poorer HRQoL in three of the four domains than those who were not worried: physical health, psychological health and environment.

Participants who were concerned about the lack of enough insufficient disinfectant supplies had poorer HRQoL in all four domains. Finally, participants who were worried about job loss due to COVID-19 had poorer HRQoL in physical health, psychological health and environment domains.

Table 2 presents the results of the independent *t*-tests. The association between the impact of COVID and HRQoL remained statistically significant in multiple linear regressions after controlling for the sociodemographic factors. Table 3 shows the results of the multiple linear regression analysis.

## 4. Discussion

Many participants were worried that they would be infected with COVID-19. There are some possible explanations. First, Hong Kong is not far from Wuhan, China, where the initial outbreak of COVID-19 is thought to have occurred [33]. Moreover, the viral outbreak occurred shortly before the Chinese New Year, when crowds of people traveled back to the hometowns or other parts of the country (including Hong Kong) to gather with their family. Hong Kong citizens worried that there would be a huge surge of new imported COVID-19 cases from other parts of China, giving rise to active community transmission within Hong Kong.

We found that the fear of infection was associated with poorer HRQoL. Fear and anxiety related to COVID-19 are known to be common stressors during the COVID-19 pandemic. Excessive stress can cause psychological and physiological disruption, leading to fatigue, poor performance, and emotional control problems [34]. A review suggests that stress affects the immune system, gastrointestinal function, and endocrine system [35]. For instance, psychological stress may interfere with the immune function through direct innervation of lymphatic tissue, through the release of hypothalamic-pituitary-adrenocortical axis and sympathetic-adrenal-medullary hormones that bind to and alter the functions of immunologically active cells, or through stress-induced behavioral changes [36]. Furthermore, psychological stress may also influence physical health through its effects on other systems. For example, psychological stress was found to impair vagal tone, leading to an increased risk of cardiovascular diseases [36,37].

In addition, the fear of acquiring COVID-19 disease has led to a significant decrease in group gatherings and trips outside the home. The public is encouraged to reduce social contact and maintain physical distance to minimize the risk of transmission among the community. In spite of the use of digital communications, social relationships could be compromised due to the lack of face-to-face social interaction. Social isolation and loneliness are also found to be closely correlated with poorer physical [38] and psychological health [20].

Our findings showed that more than 40% of the study participants often suspected themselves of having COVID-19. As most people with mild COVID-19 would experience flu-like symptoms, the most common being mild fever, cough, fatigue, and diarrhea [39,40], it is understandable that some of them would think that they were infected. Besides, the result could also be explained by Barsky and Wyshak’s (1990) model of hypochondriasis and somatosensory amplification [41]. During the COVID-19 pandemic, there is ample evidence to suggest that people suffered from stress [42], fear [26], a high level of anxiety and depression [4], and hypochondriasis. When a hypochondriac is sick, he/she may pay more attention to bodily processes and misattribute a broad range of somatic sensations and misattribute them to serious diseases, which is COVID-19 in this case [43]. Consequently, it would heighten their stress level, and in turn, affect their physical and psychological health. Nonetheless, to supplement our quantitative findings, further qualitative studies should be conducted to understand why people often suspected themselves of having COVID-19.

Amid the pandemic, people more often seek out COVID-19-related health information more frequently so as to gain better insight and understanding of the latest development [44,45]. A study in mainland China found that a higher social media exposure during COVID-19 was associated with higher odds of anxiety (odds ratio = 1.72) and a combination of depression and anxiety (odds ratio = 1.91) [46]. Furthermore, people are likely to be exposed to misleading content on social media platforms, either through disinformation or misinformation [44,47]. Their anxiety might be exacerbated when the situation is exaggerated, and consequences are overestimated. In addition, harmless bodily sensations and changes may also be misinterpreted as signs of infection, which results in distress and worsens people’s psychological and physical health [43]. This explains why we found that people who had constant suspicion of being infected with COVID-19 had poorer HRQoL, particularly in the physical and psychological domains.

Furthermore, our study found that the lack of surgical masks and disinfectant supplies at home troubled nearly 40% and 30% of participants, respectively. After the SARS outbreak in 2003, most people in Hong Kong have realized the significance of protection provided surgical masks and proper hand hygiene. Moreover, to fight COVID-19, the Hong Kong government also recommends wearing a surgical mask when taking public transport or staying in crowded places and performing hand hygiene frequently [48]. Understanding the efficacy and importance of surgical masks and disinfectants, Hong Kong people flocked to stock up on these protective and cleaning supplies during the COVID-19 pandemic. However, at the very beginning of the pandemic, there was a shortage and a general price rise for such products [4]. With panic-buying sprees, many struggled to obtain sufficient disinfectant supplies. The sight of empty shelves in stores could cause widespread consternation, and those who could not obtain enough facemasks and disinfectants might be worried that they were at higher risk of infection. In addition to the fear of contracting the virus, poorer psychosocial health could be associated with unjustified public fear. Due to fear and bias, people not wearing masks are often discriminated against in Hong Kong, as they are viewed as egocentric and inconsiderate for exposing others to the risk of infection. Therefore, people who were bothered by not having enough surgical masks and disinfectant supplies had poorer HRQoL in the physical, psychological, and environmental aspects.

Lastly, almost half of the study participants were scared about losing their job. Market instability and lockdown induced by the ongoing COVID-19 pandemic may increase the likelihood of a global economic downturn [22]. With the pandemic disrupting almost all economic activities, business plummeted in nearly all sectors, especially the hospitality, aviation, and tourism industries, which are the major pillars of Hong Kong’s economy. Many employees in Hong Kong are prone to salary reduction and dismissal. According to the statistics from the Census and Statistics Department, the seasonally adjusted unemployment rate has been surging for the past 9 months, climbing to its highest level in 15 years at 5.9% (as of May 2020) [49]. Therefore, it is reasonable to fear redundancies. Income instability and fear of dismissals could lead to economic insecurity, which is found to be associated with poorer self-rated health and a number of health issues, for example, back problems, muscular pain, overall fatigue, and insomnia [50]. Apart from these adverse impacts on physical health, uncertainty about future finance and employment may also be a source of psychological stress on people [51]. Existing studies have found that job insecurity is negatively related to overall marital and family functioning, as well as mental health [50,51,52].

### Limitations

It is prudent to highlight a number of limitations of the present study. First, the temporal relationship between COVID-19 and HRQoL cannot be explored due to the cross-sectional design. Second, all outcomes were self-reported, and the findings may be affected by reporting bias. Third, there could be sampling bias from the use of an online survey in the current study; people with lower computer literacy or lower socioeconomic status may be underrepresented in the current study. However, due to the pandemic, it is not feasible to conduct a household survey. Fourth, the study findings may not be generalizable to other populations. Differences in mortality rate, incidence rates, government policy, and even the public’s belief and attitudes toward the use of surgical masks may affect how people perceive the impact of COVID-19 on their lives and daily activities. Fifth, a generic HRQoL instrument was used in the study, which may not be specific and sensitive enough to capture the impact of COVID-19 on HRQoL. Sixth, only six self-developed items were used to capture the impacts of COVID-19 in the current study. We acknowledged that these items were not representative enough to capture the worry and fear related to COVID-19 as the pandemic evolves. Nonetheless, the items were very relevant to people in Hong Kong at that particular period of time. Seventh, other important information such as health status and the number of family members were not collected. Future studies should collect the information and explore its association with HRQoL.

## 5. Conclusions

The present study found that almost 70% of the participants were worried about contracting COVID-19. Approximately 40% of participants often suspected themselves of being infected with COVID-19 and were bothered by the lack of sufficient surgical masks and job insecurity. All these negative effects of COVID-19 were associated with poorer HRQoL across different domains. Therefore, in addition to enhancing anti-epidemic measures through physical distancing, border closure, and active surveillance of COVID-19 cases, it is equally important to implement public health and social welfare policies to diminish the adverse impact of COVID-19 on the overall well-being.

## Figures and Tables

**Table 1:** Demographics of the participants.

**Mean Age (SD)**	47.26 (15.82)
	*n* (%)
**Gender**	
Male	226 (45.20)
Female	274 (54.80)
**Marital status**	
Not currently married	164 (32.80)
Currently married	336 (67.20)
**Education status**	
Below bachelor’s degree	341 (68.20)
Bachelor’s degree or above	159 (31.80)
**Employment status**	
Having a full-time job	281 (56.20)
Having a part-time job	39 (7.80)
Students	22 (4.40)
Homemakers	65 (13.00)
Retirement	64 (12.80)
Current not working	29 (5.80)
**Monthly personal income**	
HKD$ 20,000 or below	339 (67.80)
HKD$ 20,001 or above	161 (32.20)
**The impacts of COVID-19 ^**	
(i) I feel worried that I will be infected with COVID-19.	348 (69.60)
(ii) I feel worried that my family members will be infected with COVID-19.	371 (74.20)
(iii) I feel bothered because I often suspect that I have COVID-19 symptoms.	207 (41.40)
(iv) I feel bothered because I do not have enough surgical masks.	197 (39.40)
(v) I feel bothered because I do not have enough disinfectant supplies at home.	145 (29.00)
(vi) I feel worried that I may lose my job because of COVID-19.	237 (47.40)
Mean HRQOL scores (SD) *	
Physical health	68.09 (15.16)
Psychological health	59.40 (14.95)
Social relationships	62.37 (14.20)
Environment	56.88 (14.20)

^ Study participants who answered “0 = “not at all” were coded as “no”, while study participants, who answered 1 = “several days”, 2 = “more than half the days” or 3 = “nearly every day” were coded as “yes”. * The score ranges from 0 to 100, with a high score indicating better HRQoL. Abbreviations: SD: standard deviation; HRQoL: health-related quality of life.

**Table 2:** Results of independent *t*-tests.

	**Physical Health**			**Psychological Health**		
	**No**	**Yes**		**No**	**Yes**	
	**Mean (SD)**	**Mean (SD)**	**Effect Size ^🟂^**	**Mean (SD)**	**Mean (SD)**	**Effect Size ^🟂^**
(i) I feel worried that I will be infected with COVID-19. ^	72.70 (14.39)	66.07 (15.06) **	0.45	62.36 (14.95)	58.11 (14.79) **	0.29
(ii) I feel worried that my family members will be infected with COVID-19. ^	71.46 (13.81)	66.91 (15.44) **	0.31	61.11 (15.07)	58.81 (14.88)	0.15
(iii) I feel bothered because I often suspect that I have COVID-19 symptoms. ^	72.77 (13.52)	61.46 (14.91) **	0.79	62.57 (14.99)	54.91 (13.72) **	0.53
(iv) I feel bothered because I do not have enough surgical masks. ^	71.25 (14.93)	63.22 (14.22) **	0.55	61.58 (15.78)	56.05 (12.91) **	0.38
(v) I feel bothered because I do not have enough disinfectant supplies at home. ^	71.34 (14.74)	60.12 (13.12) **	0.80	61.61 (15.27)	53.99 (12.65) **	0.54
(vi) I feel worried that I may lose my job because of COVID-19. ^	69.09 (15.83)	66.97 (14.32)	0.14	62.21 (14.82)	56.28 (14.50) **	0.40
	**Social Relationships**			**Environment**		
	**No**	**Yes**		**No**	**Yes**	
	**Mean (SD)**	**Mean (SD)**	**Effect Size ^🟂^**	**Mean (SD)**	**Mean (SD)**	**Effect Size ^🟂^**
(i) I feel worried that I will be infected with COVID-19. ^	65.52 (12.83)	60.99 (14.56) **	0.33	61.10 (13.45)	55.04 (14.14) **	0.44
(ii) I feel worried that my family members will be infected with COVID-19. ^	63.89 (14.37)	61.84 (14.12)	14.39	58.45 (13.83)	56.33 (14.30)	0.15
(iii) I feel bothered because I often suspect that I have COVID-19 symptoms. ^	63.65 (14.50)	60.55 (13.60) *	0.22	59.35 (14.87)	53.38 (12.39) **	0.44
(iv) I feel bothered because I do not have enough surgical masks. ^	63.17 (14.62)	61.13 (13.47)	0.15	58.82 (15.45)	53.90 (11.42) **	0.36
(v) I feel bothered because I do not have enough disinfectant supplies at home. ^	63.33 (14.33)	60.00 (13.63) *	0.24	58.42 (15.03)	53.13 (11.07) **	0.40
(vi) I feel worried that I may lose my job because of COVID-19. ^	62.99 (13.51)	61.67 (14.92)	0.09	59.01 (14.20)	54.52 (13.84) **	0.32

^ Study participants who answered “0 = “not at all” were coded as “no”, while study participants, who answered 1 = “several days”, 2 = “more than half the days” or 3 = “nearly every day” were coded as “yes”. The HRQOL score ranges from 0 to 100, with a high score indicating better HRQOL * *p*-value <0.05, ** *p*-value <0.01. **^🟂^** Cohen’s d effect size.

**Table 3:** Results of multiple linear regressions.

	Physical Health	Psychological Health	Social Relationships	Environment
	β	β	β	β
(i) I feel worried that I will be infected with COVID-19. (ref: no)	−7.42 **	−5.18 **	−5.46 **	−6.88 **
(ii) I feel worried that my family members will be infected with COVID-19. (ref: no)	−4.78 **	−2.88	−2.71	−2.73
(iii) I feel bothered because I often suspect that I have COVID-19 symptoms. (ref: no)	−11.20 **	−7.74 **	−3.24 *	−6.00 **
(iv) I feel bothered because I do not have enough surgical masks. (ref: no)	−7.92 **	−5.88 **	−2.41	−5.20 **
(v) I feel bothered because I do not have enough disinfectant supplies at home. (ref: no)	−10.55 **	−7.58 **	−3.31 *	−5.27 **
(vi) I feel worried that I may lose my job because of COVID-19. (ref: no)	−4.67 **	−7.34 **	−2.14	−4.97 **

All regression models were controlled for age, gender, marital status, employment status and monthly personal income. The HRQoL score ranges from 0 to 100, with a high score indicating better HRQOL. ** *p*-value <0.01, * *p*-value <0.05. ^ Study participants who answered “0 = “not at all” were coded as “no”, while study participants, who answered 1 = “several days”, 2 = “more than half the days” or 3 = “nearly every day” were coded as “yes”.

## Data Availability

The data presented in this study are available on request from the corresponding author.
